# Neuropsychological, Neurovirological and Neuroimmune Aspects of Abnormal GABAergic Transmission in HIV Infection

**DOI:** 10.1007/s11481-016-9652-2

**Published:** 2016-01-30

**Authors:** Tetyana Buzhdygan, Joshua Lisinicchia, Vipulkumar Patel, Kenneth Johnson, Volker Neugebauer, Slobodan Paessler, Kristofer Jennings, Benjamin Gelman

**Affiliations:** Department of Pathology, University of Texas Medical Branch, 301 University Blvd, 77555-0609 Galveston, TX USA; Department of Neuroscience and Cell Biology, University of Texas Medical Branch, 301 University Blvd, 77555-0609 Galveston, TX USA; Department of Pharmacology and Toxicology, University of Texas Medical Branch, 301 University Blvd, 77555-0609 Galveston, TX USA; Department of Pharmacology and Neuroscience, School of Medicine, Texas Tech University Health Sciences Center, 3601 4th Street, 79430-6592 Lubbock, TX USA; Department of Preventive Medicine and Community Health, University of Texas Medical Branch, 301 University Blvd, 77555-0609 Galveston, TX USA

**Keywords:** Autopsy, Glutamate decarboxylase, GABA, GABAergic, HIV associated neurocognitive disorders, HIV encephalitis

## Abstract

The prevalence of HIV-associated neurocognitive disorders (HAND) remains high in patients with effective suppression of virus replication by combination antiretroviral therapy (cART). Several neurotransmitter systems were reported to be abnormal in HIV-infected patients, including the inhibitory GABAergic system, which mediates fine-tuning of neuronal processing and plays an essential role in cognitive functioning. To elucidate the role of abnormal GABAergic transmission in HAND, the expression of GABAergic markers was measured in 449 human brain specimens from HIV-infected patients with and without HAND. Using real-time polymerase chain reaction, immunoblotting and immunohistochemistry we found that the GABAergic markers were significantly decreased in most sectors of cerebral neocortex, the neostriatum, and the cerebellum of HIV-infected subjects. Low GABAergic expression in frontal neocortex was correlated significantly with high expression of endothelial cell markers, dopamine receptor type 2 (*DRD2L*), and preproenkephalin (PENK) mRNAs, and with worse performance on tasks of verbal fluency. Significant associations were not found between low GABAergic mRNAs and HIV-1 RNA concentration in the brain, the history of cART, or HIV encephalitis. Pathological evidence of neurodegeneration of the affected GABAergic neurons was not present. We conclude that abnormally low expression of GABAergic markers is prevalent in HIV-1 infected patients. Interrelationships with other neurotransmitter systems including dopaminergic transmission and with endothelial cell markers lend added support to suggestions that synaptic plasticity and cerebrovascular anomalies are involved with HAND in virally suppressed patients.

**Electronic supplementary material:**

The online version of this article (doi:10.1007/s11481-016-9652-2) contains supplementary material, which is available to authorized users.

## Introduction

The prevalence of HIV-associated neurocognitive disorders (HAND) is substantial in patients treated with cART (McArthur et al. 2010). HAND was linked to the neuropathological changes produced by HIV encephalitis (HIVE) before the era of cART (Budka 1991). Data from the cART era, however, are far less supportive of the putative association between HAND and HIVE (Gelman 2015). While the frequency of HIVE declined in cART-treated patient cohorts to below 5 %, the prevalence of HAND remains as high as 50 % (McArthur et al. 2010). Neurovirological and brain gene expression data both show that the overwhelming majority of patients with HAND taking cART do not have HIVE, and they are not likely to harbor a high concentration of replicating HIV in the CNS (Gelman et al. 2012a, 2013). Thus, the pathophysiology of HAND remains poorly understood, especially when virus replication is suppressed with cART.

HIVE is associated with synaptodendritic simplification and neuronal loss in the later stages of infection (Masliah et al. 1997). Concentrations of several synaptic structural proteins are abnormal and local protein turnover in synapses may be abnormal in HIVE (Nguyen et al. 2010; Gelman and Nguyen 2010). Those aspects of the neuropathology imply that cART-treated patients might harbor subtle types of dysfunction in synaptic transmission due to synaptic plasticity, or functional changes that are not driven by morphological changes or neurodegeneration (Gelman et al. 2004, 2012b). There is neurochemical evidence that GABAergic (Koutsilieri et al. 2001; Gelman et al. 2012b), dopaminergic (Nath et al. 2000; Koutsilieri et al. 2001; Gelman et al. 2006; Gelman et al. 2012a, 2012b), cholinergic (Koutsilieri et al. 2001), glutamatergic (Ernst et al. 2010) and serotonergic (Murray 2003) neurotransmitter systems are perturbed in these patients. Decreased immunostaining of GABAergic neocortical neurons in HIVE is especially well-documented (Masliah et al. 1992, 1995; Fox et al. 1997). GABAergic markers including calbindin and somatostatin exhibit decreased immunostaining in tissue sections with HIVE (Masliah et al. 1992, 1995; Fox et al. 1997), and potential synergy between HAND and concomitant methamphetamine abuse has been suggested for these outcomes (Langford et al. 2003; Chana et al. 2006). Other evidence shows that several mRNA transcripts pertaining to GABAergic proteins are dysregulated in the dorsolateral prefrontal cortex (DLPFC) of HIVE patients (Gelman et al. 2004, 2012a). Whether disturbances in neural transmission in HIV infection represent synaptic plasticity among viable synaptic networks, versus outright neuronal death and subsequent degeneration remains controversial.

Inhibitory GABAergic interneurons constitute 20 % of the total number of neurons in the neocortex. These neurons modulate the activity of principal cells via alteration of the synchronization of excitatory activity of pyramidal neurons, and this is associated with cognitive and behavioral functions (Markram et al. 2004). Abnormal GABAergic neural transmission is present in brain specimens from patients with epilepsy, major depressive disorders and schizophrenia (Prince and Wilder 1967; Sanacora and Saricicek 2007; Hashimoto et al. 2008). Neurocognitive impairment in these diseases is driven in part by abnormal output of the DLPFC, and can lead to worse performance on tasks of abstract/executive function and verbal fluency (Volk et al. 2000; Thompson et al. 2009). The same underlying neuropsychological disorders often occur in patients with HAND (Klinkenberg et al. 2004; Iudicello et al. 2007) and could worsen and/or contribute to dysfunction of the DLPFC in patients with HAND (Woods et al. 2009).

In addition to modulating neocortical output, GABAergic interneurons play a pivotal role in neurovascular coupling, in which neurotransmitters regulate local microvascular blood flow in the neurovascular unit (Fergus and Lee 1997; Kocharyan et al. 2008). Using blood oxygen dependent contrast imaging (BOLD), several diseases exhibit disturbed regulation of cerebral blood flow, which often is linked to changes in GABAergic transmission (Northoff et al. 2007). Cerebral blood flow assessed using BOLD also is abnormal in HIV infected patients with and without HAND; the mechanism is not known and a potential role for altered GABAergic transmission has not been examined heretofore (Ances et al. 2011; Towgood et al. 2012). Thus, altered cerebral blood flow might be involved in HAND and HIV-associated changes in neurovascular coupling.

GABAergic neurons synthesize GABA via a rate-limiting reaction that is catalyzed by two glutamic acid decarboxylase (GAD) enzymes (GAD67 and GAD65, respectively). These two proteins are encoded by independently regulated genes located on chromosomes 2 and 10 (*GAD1* and *GAD2*, respectively). Both proteins are useful histological markers of GABAergic interneurons, although they exhibit unique patterns of cellular expression (Soghomonian and Martin 1998). Another marker of GABAergic interneurons is the gap junction protein connexin 36 (encoded by *GJD2* mRNA) (Hestrin and Galarreta 2005). Dendrites of GABAergic interneurons are interconnected by connexin 36 gap junctions and form complex inhibitory neural networks that provide critical modulation of frontal lobe output (Bennett and Zukin 2004; Hestrin and Galarreta 2005). Disrupting *GJD2* expression produces a loss of electrical coupling among GABAergic interneurons and leads to ineffective generation of fast synchronized oscillations in neocortical neural networks (Bennett and Zukin 2004). Clinically, the loss of GABAergic control of fast and ultrafast rhythms can produce abnormalities in neuronal processing, sensory perception, motor performance, learning, attention, and memory consolidation in brain neocortex (Bennett and Zukin 2004).

In order to better understand abnormal GABAergic transmission in HIV infected patients several issues need to be elucidated: 1) It is not known whether replicating HIV in the brain drives GABAergic changes, or whether suppressing virus replication with cART normalizes the change; 2) Although HAND without HIVE is the dominant clinicopathological sequence in virally suppressed patients (Gelman et al. 2012a; Gelman 2015), the importance of GABAergic anomalies in these patients and the role of HIVE need to be elucidated; 3) It is not clear what type of neuropsychological dysfunction, if any, is associated with GABAergic changes in HAND. 4) It remains unclear whether loss of GABAergic marker protein represents the death of inhibitory neurons (i.e., classical pathological neurodegeneration) as is often suggested, or instead reflects modified expression of GABAergic marker proteins in viable interneurons (i.e., accommodation due to synaptic plasticity) (Akbarian et al. 1995; Volk et al. 2000; Gelman et al. 2006). 5) Basic brain regional anatomy and circuit-level dysfunction of GABAergic inhibitory networks need to be better characterized in HIV infected subjects (Gelman et al. 2012a). 6) Because of the role of GABA in regulating cerebral blood flow, it is not known whether GABAergic anomalies contribute to abnormal neurovascular biology in HIV infected patients (Strazza et al. 2011). To address these issues we evaluated neurochemical markers of GABAergic transmission in 449 brain specimens obtained from HIV-infected subjects, many of whom underwent antemortem neuropsychological testing.

## Materials and Methods

### Human Brain Specimens

GABAergic mRNA concentrations were measured in the dorsolateral prefrontal cortex (DLPFC) of 515 human post-mortem brain specimens obtained from National NeuroAIDS Tissue consortium (NNTC) (Morgello et al. 2001). This particular patient cohort was described in a previous communication (Gelman et al. 2012b). DLPFC is of prime relevance because the functional output of this brain sector is abnormal in HAND (Woods et al. 2009). 449 of the patients were infected with HIV-1 and 66 were demographically comparable HIV seronegative decedents (Gelman et al. 2006, 2012b) (Table S1). 131 out of 449 of the HIV-infected patients died before 1997 and/or before cART was introduced to the patient. cART status was defined as being active if the patient had taken at least 2 nucleoside/nucleotide reverse transcriptase inhibitors (NRTIs) or 1 nonnucleoside reverse transcriptase inhibitor (NNRTI) and 1 protease inhibitor within 1 year of death (Gelman et al. 2013). 290 out of 313 patients with documented cART status were cART active within 1 year of death. 219 out of 449 of the HIV-infected patients participated in longitudinal clinical studies after 1999 during the cART era, which included neuropsychological testing in the 6 months before death. For the subjects who were studied clinically, written consent was obtained for subjects at four collection sites in the USA. The following offices maintained institutional review boards (IRBs) that provided oversight for the protection of human subjects: 1) The University of Texas Medical Branch Office of Research Subject Protections; 2) Mount Sinai Medical Center Program for the Protection of Human Subjects; 3) University of California, San Diego Human Research Protections Program; 4) University of California, Los Angeles Office of the Human Research Protection Program.

### Neuropsychological Testing and Substance use Survey

Neuropsychological evaluations and substance use surveys were performed every 6 months using the protocol of the National NeuroAIDS Tissue Network (NNTC). The NNTC neurocognitive test battery included assessment of seven cognitive domains: 1) verbal fluency (Controlled Oral Word Association Test, COWAT- FAS); 2) speed of information processing (Wechsler adult intelligence scale, WAIS-III Digit Symbol and Symbol Search subtest and Trail Making Test Part A); 3) learning (Hopkins Verbal Learning Test – Revised, HVLT–R; Total Trial 1–3 Recall and Brief Visuospatial Memory Test – Revised, BVMT–R, Total Trial 1–3 Recall); 4) memory (HVLT–R Delayed Recall, BVMT–R Delayed Recall); 5) executive functions (Wisconsin Card Sorting Test, WCST 64-item version; perseverative responses and TMT Part B) (6) attention and work- ing memory (WAIS-III Letter-Number Sequencing, PASAT-50); 7) motor (Grooved Pegboard Test, dominant and nondominant hand performances). The Wide Range Achievement Test-3 (WRAT-3) Reading subtest was administered as an estimate of premorbid verbal intellectual functioning (Woods et al. 2004). The Psychiatric Research Interview for Substance and Mental Disorders (PRISM) or the Composite International Diagnostic Interview (CIDI) was used to obtain self-reported lifetime histories of substance abuse and dependence (Robins et al. 1988; Morgello et al. 2001). A diagnosis of HAND was assigned guided by American Academy of Neurology criteria as modified by the Frascati Criteria.

### Brain Specimen Dissection and Neuropathological Data

Brain specimens obtained fresh at autopsy were bisected in the sagittal plane. One hemisphere was sliced fresh and the slices were stored at – 80 °C. The other hemisphere was immersed in a 20 % formalin solution at 4 °C for 10 days. 200–300 mg of frozen grey matter from the dorsolateral prefrontal cortex in Brodmann area 9 or 8 was dissected for mRNA and protein extraction. Other brain regions were dissected for regional comparisons as indicated in the figures and tables. Frozen samples were kept on dry ice and placed in pre-weighed and pre-cooled vials and were stored at – 80 °C. Specimens were assayed in batches that were freshly thawed. The neuropathological diagnoses were obtained using the brain sampling and staining protocols of the NNTC (Morgello et al. 2001). The diagnosis of HIV encephalitis (HIVE) was made according to established criteria (Budka 1991; Gelman et al. 2013).

### Quantitative Real-Time PCR

About 100 mg of brain tissue was homogenized in 1 ml of QIAzol reagent (RNeasy Mini Kit, Qiagen, Valencia, CA) for isolation of total RNA according to the standard manufacturer’s protocol. Single strand cDNA was prepared using Bio-Rad iScript cDNA synthesis Kit (Bio-Rad, Hercules, CA). 2 ug of brain mRNA were mixed with 8 ul of 5x iScript reaction mix, 2 ul of iScript reverse transcriptase and total volume was adjusted to 40 ul with nuclease-free water. The reaction mixture was incubated in the Bio-Rad I-cycler programmed for 5 min at 25 °C, 30 min at 42 °C, 5 min at 85 °C and held at 4 °C. For quantitation of the mRNA primers and FAM probes were obtained for *GAD1* (Cat. No. Hs01065893_m1), *GAD2* (Cat. No. Hs00609534_m1), *GJD2* (Cat. No. Hs00706940_s1), *ISG15* (Cat. No. Hs00192713_m1), *MX1* (Cat.No. HS00182073_m1), *IRF1* (Cat.No. Hs00971959_m1), *GZMB* (Cat.No. Hs01554355_m1), *CD4* (Cat.No. Hs01058407_m1), *CD8A* (Cat.No. Hs01555600_m1), *CD19* (Cat.No. Hs99999192_m1), *CD68* (Cat.No. Hs00154355_m1), *CD163* (Cat.No. Hs01016661_m1), *VWF* (Cat.No. Hs00169795_m1), *PECAM1* (Cat.No. Hs00169777_m1), from Applied Biosystems, Foster City, CA, USA), Glyceraldehyde 3-phosphate dehydrogenase (*GAPDH*, Cat. No. Hs99999905_m1) was used as the normalizing gene. For a 20 ul reaction mixture 1ul of cDNA was mixed with 10 ul of TaqMan 2x PCR Master Mix (Applied Biosystems), 8 ul H2O and 1 ul ABI 20x primer (*gene of interest* or *GAPDH*). PCR reactions were performed on Mastercycler RealPlex (Eppendorf, Germany) under the following conditions: activation (10 min at 95 °C), and 40 cycles of denaturation (15 s at 95 °C) – annealing/extension (60 s at 60 °C). Experiments were performed on 96-well plates, each sample was run in duplicates, triple negative controls and standard calibrators were run on each plate. Duplicate C_T_ values were analyzed using the comparative C_T_ (ΔΔC_T_) method as described by the manufacturer (Applied Biosystems). The amount of targets (2^-ΔΔCT^) was obtained by normalizing to endogenous reference (*GAPDH*).

### Immunoblotting

Frozen tissue samples were homogenized in 3x volume of buffer (10 mM Tris–HCl, 5 mM MgCl_2_, 0.5 mM dithiothreitol (DTT), 0.03 % Triton X-100, pH 7.8) by silica beads beating (2 times for 20 s) and sonication (2 times for 20 s) with tubes kept on ice for 5 min between sessions. Protein concentration was determined using Bio-Rad Protein Assay (Bio-Rad Laboratories, Hercules, CA) and bovine serum albumin (BSA) standards. 15 ug of total protein were loaded into Criterion Precast 18-well 4–20 % gradient Tris–HCl gel (Bio-Rad Laboratories, Hercules, CA) for SDS-PAGE electrophoresis and run at 180 volts for 45 min. Separated proteins were transferred to PVDF membrane (Amersham Biosciences, Piscataway, NJ) in 10 mM Tris-glycine buffer containing 10 % methanol at 75 W for 1 h at 4 °C. The membrane was blocked in 5 % non-fat milk in Tris buffer Saline (TBS-T; 50 mM Tris–HCl, 150 mM NaCl, 0.01 % Tween-20) for 1 h and incubated with mouse anti-GAD67 (Cat. No. ab22050, Abcam) or mouse anti-GAPDH (Cat.No. sc-47724, Santa Cruz Biotechnologies, Santa Cruz, CA, USA) primary antibodies diluted in blocking solution overnight at 4 °C. The membrane was washed three times in TBS-T and incubated with sheep anti-mouse IgG antibodies (Cat. No. Na931V, GE Healthcare, UK) diluted 1:1000 in blocking solution for 2 h. Then the membrane was incubated with Enhanced Chemiluminescence Detection Reagent (Amersham Biosciences, Piscataway, NJ) for 2 min and exposed to Kodak BioMax XAR film (Kodak, Rochester, NY). Film was developed and scanned and bands densities were measured using ImageJ software (NIH, Bethesda, MD, USA).

### Immunohistochemistry

After formalin fixation, blocks of brain tissue were trimmed and embedded in paraffin wax. Sections of tissue (6–8 um) were mounted on glass slides (Superfrost Plus Gold, Erie Scientific, Portsmouth, NJ). The sections were deparaffinized in xylene, rehydrated in graded alcohols and immersed in 0.01 M sodium citrate buffer heated in a microwave oven for 20 min. Unspecific binding was blocked for 20 min with 1 % normal serum and then the sections were incubated overnight at 4 °C with primary antibodies diluted in blocking solution. Following primary antibodies were used: anti-GAD67 (Cat. No. ab26116, Abcam, Cambridge Science Park, Cambridge, England) diluted 1:1000, anti-CD31 (Cat. No. M0823, Dako, Via Real, Carpinteria, CA, USA) diluted 1:30, anti-granzyme B (Cat.No. GB-7, Sanquin, Sanquin Blood Supply, Amsterdam, Netherlands) diluted 1:50, anti-CD8A (Cat.No. M710301-2, Dako) diluted 1:50, anti-CD68 (Cat.No. M0814, Dako) diluted 1:50. Next day tissue was treated with 3 % hydrogen peroxide for 10 min and rinsed with TBS-T (50 mM Tris–HCl, 150 mM NaCl, 0.05 % Tween-20). Appropriate anti-mouse or anti-rabbit Vectastain secondary antibodies were applied for 1 h, followed by Vectastain ABC and Vectastain DAB color development using peroxidase-diaminobenzidine reactions (Vector Laboratories, Burlingame, CA). Sections were dehydrated and mounted using Permount (Fisher Scientific, Hampton, NH). Composite microscopic fields were acquired by combining contiguous images taken using an Olympus DP71 camera and Olympus DP controller software (Olympus America Inc, Center Valley, PA, USA).

### Immunofluorescence

Sections for immunofluorescence underwent deparaffinization and antigen retrieval treatment as described above. After antigen retrieval, tissue was permeabilized for 15 min with 0.1 % Triton-X in Phosphate buffer saline (PBS) and treated with ImageT FX signal enhancer (Invitrogen Molecular Probes, Eugene, Oregon) for 30 min. Unspecific binding was blocked for 1 h with 1 % normal serum and then the sections were incubated overnight at 4 °C with primary antibodies diluted in blocking solution. Primary antibodies were used: mouse anti-GAD67 (Cat. No. mab5406, EMD Millipore Corporation, Billerica, MA, USA) diluted 1:100, rabbit anti-parvalbumin (Cat. No. ab11427, Abcam) diluted 1:1000, mouse anti-dopamine receptor 2 (Cat.No. sc-5303, Santa Cruz Biotechnologies, Santa Cruz, CA, USA) diluted 1:50. Next day sections were rinsed in with TBS-T (50 mM Tris–HCl, 150 mM NaCl, 0.05 % Tween-20) and mixture of secondary antibodies diluted 1:500 in PBS were applied for 3 hs (AlexaFluor 488 goat anti-mouse (Cat.No. A11029), and AlexaFluor 594 goat anti-rabbit (Cat.No.A11037), Life Technologies, Grand Island, NY, USA). After incubation with secondary antibodies, sections were rinsed with TBS-T and treated with Sudan Black B (0.5 in 70 % ethanol) for 20 min. Then sections were rinsed in ddH2O, air dried and mounted using ProLong Gold with DAPI mounting media (Life Technologies). Confocal images were then acquired with a Zeiss LSM-510 Meta confocal microscope with 63x / 1.40 Oil DIC (WD = 0.19 mm) objective (Carl Zeiss Microscopy, Peabody, MA, USA) and processed using LSM Image Browser (Zeiss).

### Statistical Analysis

Statistical analysis was performed using Microsoft Excel 2010 (Microsoft Corporation, Redmond, Washington, DC, USA), and GraphPad 6 (GraphPad Software, Inc., La Jolla, CA, USA). Two-tailed Student’s *t*-test was used to compare two groups. One-way analysis of variance with post hoc Scheffé’s or uncorrected Fisher’s LSD tests were used to compare the mean of one group to the mean of other groups. Pearson’s correlation was used to analyze correlations between GABAergic mRNAs level and neurovirological, neuroimmunological and neurocognitive measurements. These data were log_10_ transformed to normalize the distributions. Significance of correlation was corrected for the false discovery rate using the Bonferroni correction. Significance was set at α = 0.05. Fisher z-transformation was used to estimate the significance of the difference between two correlation coefficients found in two independent samples. Multiple regression models were used to determine whether potentially confounding factors such as age, gender, race, and drugs of abuse affected GABAergic mRNA.

## Results

### GAD1, GAD2 and GJD2 mRNA Expression

**Fig. 1:**
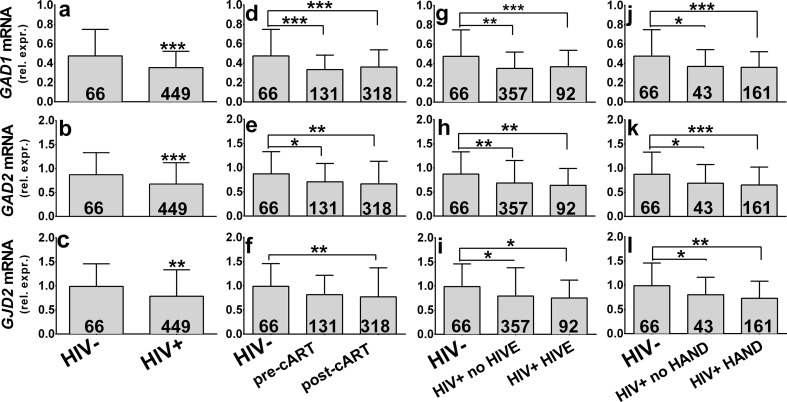
Glutamate decarboxylase 1 (*GAD1*), glutamate decarboxylase 2 (*GAD2*), and connexin 36 (*GJD2*) mRNAs in the dorsolateral prefrontal cortex of 449 HIV infected and 66 HIV seronegative patients (Panels a - l). “Rel. exp.” on the ordinates denotes mRNA expression relative to *GAPDH* mRNA. The number of subjects in each group is given in the bars. Panels a - c. GABAergic mRNA expression was significantly lower in the HIV infected patients. Panels d – f. HIV-positive subjects were divided into subjects who died before and after the introduction of cART. mRNA expression was significantly lower in pre-cART and post-cART HIV patients as compared to the uninfected comparison group. There were no significant differences between pre-cART and post-cART groups. Panels g - i. HIV-positive subjects were divided according to neuropathological diagnosis of HIV-encephalitis (HIVE). All three mRNAs were significantly lower in patients without HIVE and with HIVE when compared to the uninfected comparison group. There were no significant differences between HIVE and no HIVE groups. Panels j - l. HIV-positive subjects were divided according to the diagnosis of HIV-associated neurocognitive disorders (HAND). Compared to the uninfected comparison group, all three mRNAs were significantly lower in patients without HAND and with HAND. There were no significant differences between HAND and no HAND groups. Mean ± standard deviation. *p < 0.05; **p < 0.01; ***p < 0.001

Three markers of GABAergic interneurons in the DLPFC were abnormally expressed in the HIV-infected patients compared to normal uninfected controls. mRNAs that correspond to GAD67, GAD65, and connexin 36 proteins all were significantly lower in the HIV positive subjects versus the HIV negative controls (for *GAD1*, -26 % and *p* < 10^−4^, for *GAD2*, -22 % and *p* < 0.001, for *GJD2*, -21 % and *p* = 0.0043; Fig. 1a–c). When HIV-infected subjects were divided into those that died before the era of cART versus after, all three mRNAs were significantly lower in both eras (by 30 and 24 % for *GAD1*, 19 and 24 % for *GAD2*, 17 and 22 % for *GJD2*; Fig. 1d–f). Between group analysis using one-way analysis of variance (ANOVA) and post hoc Scheffé’s test yielded F = 13.27 and *p* < 10^−4^ for *GAD1*, F = 5.957 and *p* = 0.0028 for *GAD2*, and F = 4.425 and *p* = 0.012 for *GJD2*. The pre- and post-cART era groups did not differ significantly from each other. When HIV-positive subjects were sorted according to whether or not they had HIV encephalitis (HIVE) at autopsy, the mRNAs were significantly lower than HIV-negatives in the patients without and with HIVE both (by 26 and 23 % for *GAD1*, 21 and 27 % for *GAD2*, 20 and 24 % for *GJD2*; Fig.1g–i). Between group analysis using one-way analysis of variance (ANOVA) and post hoc Scheffé’s test yielded F = 12.55 and *p* < 10^−4^ for *GAD1*, F = 5.960 and *p* = 0.0028 for *GAD2* and F = 4.313 and *p* = 0.014 for *GJD2*. The groups with and without HIVE did not differ from each other significantly with respect to these mRNAs.

### Regional Distribution of the GABAergic Anomaly in the CNS

**Fig. 2:**
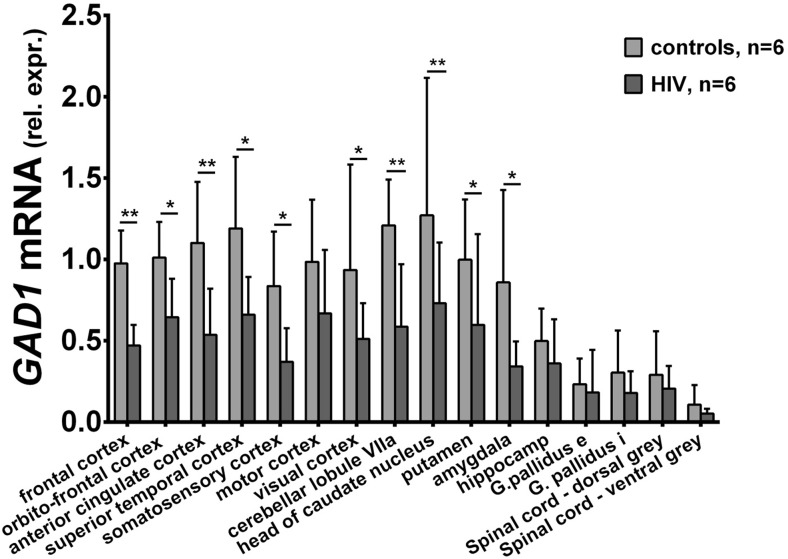
Glutamate decarboxylase 1 mRNA (*GAD1*) expression was measured in 16 different brain regions obtained from 6 HIV-infected patients and 6 uninfected patients. Using two-way ANOVA, only variation in *GAD1* mRNA concentrations between brain regions was significant (F (16, 218) = 7.17, *p* < 10^−4^). *GAD1* was lower in the HIV infected brain specimens in most regions including neocortex, cerebellum and neostriatum (caudate nucleus and putamen). Uncorrected Fisher’s LSD tests were significant for dorsolateral prefrontal (- 51.7 %, *p* = 0.010), anterior cingulate (- 51.2 %, *p* = 0.009), superior temporal (- 44.5 %, *p* = 0.013), somatosensory (- 55.6 %, *p* = 0.042), and visual cortices (- 45.4 %, *p* = 0.038), cerebellar lobule VIIa (- 51.5 %, *p* = 0.003), head of caudate nucleus (- 42.5 %, *p* = 0.009), putamen (- 40.1 %, *p* = 0.049), and amygdala (- 60 %, *p* = 0.012). Mean decrease of *GAD1* mRNA expression in the orbitofrontal cortex was - 36.2 % and almost reached significance (*p* = 0.0728). Spinal cord, paleostriatum, and hippocampus also had slightly lower *GAD1* in the HIV infected brain specimens, but those differences were not significant statistically. Relative expression of *GAD1* mRNA was normalized to *GAPDH* mRNA. Mean ± standard deviation. *p < 0.05; **p < 0.01; ***p < 0.001

To determine if specific brain regions were selectively vulnerable to having low GABAergic markers, we measured *GAD1* mRNA in a total of 16 sectors of the human CNS, including 7 neocortical regions, 6 subcortical regions, cerebellum and spinal cord (Fig. 2). Six HIV negative controls and 6 HIV-infected subjects were selected for study based upon their GABAergic mRNA values in the DLPFC. There was substantial variation in *GAD1* mRNA expression between brain regions (F (16, 218) = 7.17, *p* < 10^−4^, two-way ANOVA). In almost all regions *GAD1* mRNA was lower in the HIV infected subjects, including neocortex, neostriatum and cerebellar lobule. Exceptions were spinal cord, paleostriatum (globus pallidus) and hippocampus in which the lower *GAD1* expression was not statistically significant.

### Relationship of GABAergic mRNAs to Demographic Factors and Substance use History

Some studies have suggested that substance abuse can produce changes in GABAergic transmission (Zhang et al. 2006; Satta et al. 2008). When sorted according to self-reported history of drug abuse, HIV-infected subjects with and without drug abuse history contained significantly lower values for all three GABAergic markers mRNAs compared to controls (by 25 and 28 % for *GAD1*, 24 and 27 % for *GAD2*, 25 and 27 % for *GJD2*; Fig. S1 a–c) . Between group analysis using one-way ANOVA yielded F = 9.242 and *p* < 10^−4^ for *GAD1*, F = 7.124 and *p* = 0.001 for *GAD2*, and F = 8.12 and *p* = 0.0004 for *GJD2*. Between-group comparisons revealed no difference between subjects with and without a history of drug abuse.

Several CNS conditions can influence the expression of the above transcripts. To examine whether such confounders impact the concentration of GABAergic mRNAs, HIV-positive subjects were sorted according to whether or not they had comorbid CNS disease (such as progressive multifocal leukoencephalopathy (PML), primary CNS Lymphoma (PCNSL), CMV ventriculoencephalitis (CMVE), toxoplasmosis Encephalitis (TE), meningitis (such as aseptic leptomeningitis, bacterial leptomeningitis, cryptococcal meningitis), or hemorrhages). All three mRNAs were significantly lower in both groups (without and with comorbidities) of HIV-positives (by 25 and 28 % for *GAD1*, 22 and 29 % for *GAD2*, 23 and 22 % for *GJD2*; Fig. S3 g - i). Between group analysis using one-way analysis of variance (ANOVA) and post hoc Tukey’s test yielded F = 12.34 and *p* < 10^−4^ for *GAD1*, F = 8.418 and *p* = 0.0003 for *GAD2* and F = 9.648 and *p* < 10^−4^ for *GJD2*. The groups without and with comorbid CNS diseases tissue did not differ from each other significantly with respect to these mRNAs.

Age, gender and ethnicity are other potential variables that could affect GABAergic genes expression in human brain specimens (Pinto et al. 2010; Seney et al. 2013). Multiple regression models showed that potentially confounding co-variables including drug abuse, age, gender, and race were not significantly related to *GAD1* and *GJD2* mRNAs (not shown). For *GAD2* mRNA statistical models suggested a significantly higher level in males (*p* = 0.0027), but overall results for *GAD2* were not changed when the results were corrected statistically for the influence of gender (data not shown).

### Relationship Between GABAergic mRNAs, Clinical Virology and Neurovirology

Correlation coefficients between brain HIV RNA loads and GABAergic mRNAs in the DLPFC were not significant (Table S2). When GABAergic mRNAs were correlated with HIV RNA in blood plasma, the correlation coefficient was negative and statistically significant for *GAD2* mRNA (*n* = 264, r = - 0.1372, *p* = 0.0258), but not the other mRNAs. When GABAergic mRNAs were correlated with HIV RNA in the CSF samples that were available, correlation coefficients were negative and significant for two GABAergic mRNAs (*GAD2*: *n* = 191, r = -0.1582, *p* = 0.0286; *GJD2*: *n* = 191, r = - 0.1801, *p* = 0.0127). Neurovirological correlations in patients with HIVE often differ from those without HIVE (Gelman et al. 2012a, 2013). To determine whether HIVE influenced the neurovirological correlation with GABAergic mRNAs, patients with and without HIVE were evaluated separately. *GAD1* mRNA was negatively and significantly correlated with brain HIV RNA in the subjects without HIVE, whereas the correlation for patients with HIVE had a positive coefficient that almost reached significance (Table S2). Fisher r to Z transformation showed that the two oppositely sloped regression lines were statistically different, comparing patients with HIVE versus without it (two-tailed *p* = 0.0278). These results suggest that HIVE, which produces high loads of replicating HIV in the brain and increased inflammation, tends to blunt the decrease of the GABAergic mRNAs in HIV infected patients.

When HIV-infected subjects were divided into those that have low versus high viral load in the cerebrospinal fluid (CSF), all three mRNAs were significantly lower in both groups of HIV-positives (by 22 and 36 % for *GAD1*, 19 and 36 % for *GAD2*, 22 and 36 % for *GJD2*; Fig. S3 a - c). Between group analysis using one-way analysis of variance (ANOVA) and post hoc Tukey’s test yielded F = 12.44 and *p* < 10^−4^ for *GAD1*, F = 10.33 and *p* < 10^−4^ for *GAD2*, and F = 14.37 and *p* < 10^−4^ for *GJD2*. The low and high viral load in the CSF groups did not differ significantly from each other. When HIV-positive subjects were sorted according to low versus high viral load in the frontocortical grey matter, all three mRNAs were significantly lower in both groups of HIV-positives (by 27 and 25 % for *GAD1*, 20 and 23 % for *GAD2*, 22 and 24 % for *GJD2*; Fig. S3 d - f). Between group analysis using one-way analysis of variance (ANOVA) and post hoc Tukey’s test yielded F = 12.29 and *p* < 10^−4^ for *GAD1*, F = 5.673 and *p* = 0.0037 for *GAD2* and F = 9.642 and *p* < 10^−4^ for *GJD2*. The groups with low versus high viral load in brain tissue did not differ from each other significantly with respect to these mRNAs.

### Relationship of GABAergic mRNAs to Neurocognitive Impairment

Abnormal GABAergic transmission occurs in diseases that can adversely affect neurocognitive test performance, especially on tasks that are driven by the DLPFC (Volk et al. 2000; Woods et al. 2009). To address whether the GABA disturbance was related to HIV-associated neurocognitive disorders (HAND), we sorted the subjects according to the diagnosis of HAND and the GABAergic mRNAs were compared in the two groups. All three GABAergic mRNAs were significantly lower in the subjects with HAND relative to the uninfected controls; the same result was obtained in the subjects without HAND (Fig. 1j–l). Between group analysis using one-way analysis of variance (ANOVA) and Scheffé’s post hoc test yielded F = 8.420 and *p* = 0.0003 for *GAD1*, F = 7.384 and *p* < 0.0008 for *GAD2*, and F = 10.51 and *p* < 10^−4^ for *GJD2*. There were no significant differences between HAND and no HAND groups. When subjects with HAND were sorted according to the severity of impairment, all three mRNAs were significantly lower in patients diagnosed with HIV-associated dementia (HAD) and Mild Neurocognitive Disorder (MND) compared to seronegative controls (by 24 and 18 % for *GAD1*, 32 and 22 % for *GAD2*, 29 and 22 % for *GJD2*; Fig. S1 d–f). One-way analysis of variance (ANOVA) yielded F = 5.294 and *p* = 0.0015 for *GAD1*, F = 6.688 and *p* = 0.0002 for *GAD2*, and F = 7.215 and *p* = 0.0001 for *GJD2*. Groups of HIV-positives with different degrees of cognitive impairment did not differ from each other. There were no significant differences between HIV-patients diagnosed with Asymptomatic Neurocognitive Impairment (ANI) and controls.

Using the composite neurocognitive impairment T score, Pearson’s correlation analysis showed no significant correlation with GABAergic mRNAs in the DLPFC. When GABAergic mRNAs were compared to performance in distinct functional domains that were assessed in the test battery, there was a significant correlation between low GABAergic mRNAs and worse performance on the verbal fluency task (*p* = 0.0036 for *GAD1* mRNA, *p* = 0.0005 for *GAD2* mRNA, *p* = 0.0013 for *GJD2* mRNA) after correction for multiple comparisons (Table 1).

**Table 1:** GABAergic transcripts correlated with normalized neurocognitive T-scores

*HIV*+		*GAD1* mRNA		*GAD2* mRNA		*GJD2* mRNA	
Neurocognitive test domain	*n*	*r*	*p*	*r*	*p*	*r*	*p*
Memory	218	0.0322	0.6363	0.0256	0.7070	0.0528	0.438
Attention and working memory	212	0.0229	0.7403	0.0791	0.2515	0.0493	0.475
Learning	219	0.0073	0.9145	0.0653	0.3361	0.0539	0.4274
Motor	202	0.0353	0.6180	0.0306	0.6650	0.1124	0.1112
Verbal fluency	216	0.1974	0.0036*	0.2358	0.0005*	0.2171	0.0013*
Abstract executive	209	0.0614	0.3771	0.0974	0.1606	0.0918	0.1861
Speed of information processing	217	0.0377	0.5810	0.0375	0.5827	0.0461	0.4993
Global T-Score	196	0.0793	0.2690	0.1003	0.1619	0.1072	0.135

*Asterisk denotes statistically significant p value. GABAergic transcripts were measured in the frontal neocortex

HIV human immunodeficiency virus type 1, HIVE HIV encephalitis, r correlation coefficient

### Relationships Between GABAergic mRNAs, Endothelial and Immune Cell Markers

**Fig. 3:**
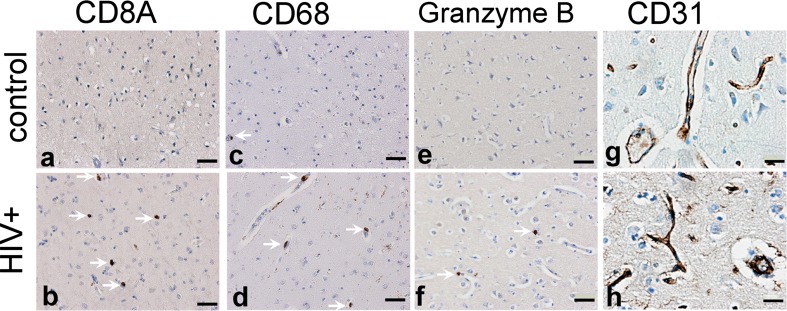
Neuroimmune and endothelial cells were immunostained in the dorsolateral prefrontal cortex of the HIV-seronegative control (panels **a**, **c**, **e**, **g**) and HIV-infected subjects (**b**, **d**, **f**, **h**). CD8+ cytotoxic T cells (*arrows*, **a** vs **b**) and CD68+ macrophages (*arrows*, **c** vs **d**) were more numerous in specimens from HIV-infected subjects with low *GAD1* expression, as suggested by concentrations of the corresponding mRNAs (see Table 2). Granzyme B-containing inflammatory cells were more numerous in specimens from HIV- infected subjects with low *GAD1* expression (arrows, **e** vs **f**). Staining for the endothelial cell marker CD31 produced more intense marking of blood vessel walls (*arrows*) in HIV- infected subjects with low *GAD1* (g vs h). *Bars* represent 50 um in **a** – **f**, and 20 um in **g** and **h**

In addition to the potential influence of CNS virus replication, it has been suggested that neuroimmune anomalies including changes in the neurovascular unit (Strazza et al. 2011) and heightened interferon responses could drive HAND (Gelman et al. 2012a). When mRNAs that mark neurovascular and neuroimmune type changes were examined, strong correlations were found between low GABAergic mRNAs and high expression of the endothelial cell markers *PECAM1* and *VWF* (Table 2). Low GABAergic mRNAs were correlated significantly, but less strongly with high expression of neuroimmune type markers including a prototypical type 1 interferon response gene (MX1), a type 2 interferon response gene (IRF1), macrophages (CD163 and CD68), CD8+ cytotoxic T lymphocytes (CD8A), and natural killer cells (GZMB). In contrast, markers for B lymphocytes (CD19) and CD4+ T lymphocytes (CD4) were not significantly correlated with low GABAergic mRNAs. Immunohistochemical staining of brain tissue for marker antigens corresponding to endothelial cells, macrophages, CD8+ lymphocytes, and natural killer cells generally confirmed these associations at protein and cellular levels (Fig. 3).

**Table 2:** GABAergic transcripts correlated with neuroimmunological and endothelial markers

HIV+ (*n* = *449*)	*GAD1* mRNA		*GAD2* mRNA		*GJD2* mRNA	
	*r*	*p*	*r*	*p*	*r*	*p*
*MX1 mRNA*	−0.1375	0.0035*	−0.1721	0.0002*	−0.0465	0.3256
*ISG15* mRNA	−0.0306	0.5178	−0.0510	0.2809	−0.0186	0.6943
*IRF1* mRNA	−0.1574	0.0008*	−0.1504	0.0014*	−0.0804	0.0888
*CD68* mRNA	−0.1946	0.0000*	−0.1732	0.0002*	−0.1181	0.0123*
*CD163* mRNA	−0.1950	0.0000*	−0.1081	0.0221*	−0.0750	0.0563
*CD8A* mRNA	−0.1197	0.0112*	−0.1821	0.0001*	−0.0369	0.4354
*CD4* mRNA	−0.0808	0.3426	−0.0647	0.4476	0.0854	0.3157
*CD19* mRNA	−0.0501	0.2895	−0.0661	0.1620	−0.0173	0.7145
*GZMB* mRNA	−0.1450	0.0021*	−0.1553	0.0010*	−0.1018	0.0310*
*VWF* mRNA	−0.2769	0.0000*	−0.2902	0.0000*	−0.0501	0.2895
*PECAM1* mRNA	−0.3419	0.0000*	−0.3529	0.0000*	−0.1980	0.0000*
*PENK mRNA*	0.2968	0.0000*	0.3534	0.0000*	0.2018	0.0000*

*Asterisk denotes statistically significant *p* value. GABAergic, neuroimmunological and endothelial transcripts were measured in the frontal neocortex

HIV human immunodeficiency virus type 1, *r* correlation coefficient

### Interrelationships Between GABAergic, Dopaminergic and Enkephalinergic mRNAs

Dopaminergic and enkephalinergic neural transmission both have been shown to be neurochemically abnormal in the DLPFC of this HIV infected autopsy cohort (Gelman et al. 2012b). These neurotransmitter type changes were compared to the results for GABAergic mRNAs (Fig. S2). In seronegative controls *GAD1* mRNA was *positively* and significantly correlated with the transcript of a key dopaminergic receptor (*DRD2L*) that was previously shown to be regulated in the DLPFC of HIV infected patients (*n* = 66, r = 0.267, *p* = 0.03). In contrast, the same correlation analysis in the HIV infected subjects produced a significant *negative* correlation coefficient (*n* = 449, r = - 0.157, *p* = 0.0008). Using bootstrapping and Fisher z-transformations these correlations were significantly different from each other (two-tailed *p* = 0.011). Thus, reduced expression of GABAergic markers is significantly related to higher *DRD2L* expression in the HIV infected subjects but not in seronegative controls. When HIV-positive subjects were grouped according to the clinical diagnosis of HAND versus no HAND there was a significant correlation between GABAergic mRNAs and *DRD2L* in the subjects with HAND (*n* = 197, r = - 0.183, *p* = 0.01). Infected subjects without HAND also exhibited a negatively sloped regression line, but the correlation was not significant statistically (*n* = 43, r = - 0.159, *p* = 0.306), probably due to the more limited number of available subjects in that subgroup.

Preproenkephalin mRNA (*PENK*) was shown previously to be lower in HIV infected subjects in this patient cohort (Gelman et al. 2012b). Lower *PENK* was correlated significantly with low GABAergic expression in HIV infected patients (Table 2).

### GAD67 Protein Expression and Tissue Localization

**Fig. 4:**
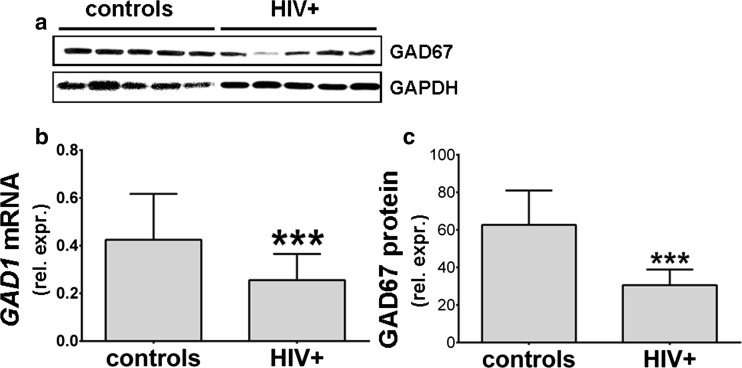
Glutamate decarboxylase 67 (GAD67) protein and mRNA expression in the dorsolateral prefrontal cortex. GAD67 immunoblotting was done on 36 HIV-infected subjects and 12 seronegative controls. GAPDH blots were done for loading controls. Equal amounts of protein were loaded to each well. GAD67 band intensities were significantly lower in 36 HIV+ subjects compared to 12 HIV- subjects (-51.2 %, *p* = 0.0001, panel c). Band intensities for ten representative subjects are illustrated in panel a. *GAD1* mRNA expression is shown for comparison (b), mean *GAD1* mRNA decrease is -39,8 %, (*p* = 0.001). GAD67 is expressed as relative to the GAPDH band intensity. *GAD1* is expressed as relative to *GAPDH* mRNA. Mean ± standard deviation is shown. P values were obtained using the Student’s *t* test. *p < 0.05; **p < 0.01; ***p < 0.001

**Fig. 5:**
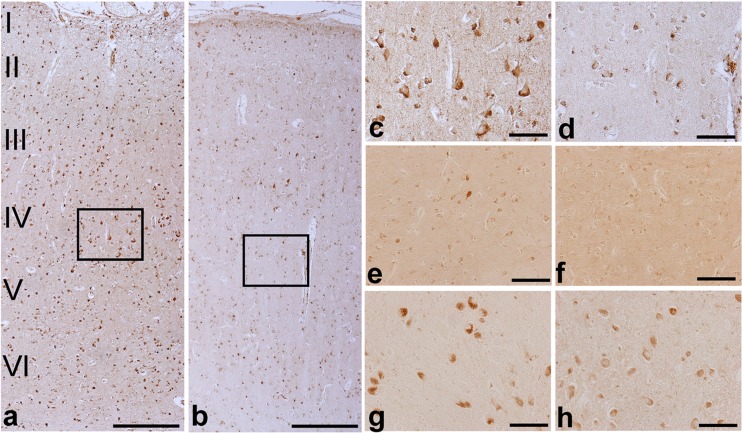
Glutamate decarboxylase 67 (GAD67) immunoreactivity in brain tissue from an HIV infected patient (**b**, **d**, **f**, **h**) and a seronegative control (**a**, **c**, **e**, **g**). Panels a and b illustrate dorsolateral prefrontal cortex (DLPFC) with neocortical laminae labeled I through VI at left. The specimen from the HIV infected subject (b) contains less intense staining in all of the laminae compared to the seronegative patient (a). Boxed areas in a and b are magnified in c and d and illustrate less intense immunostaining in pyramidal cell cytoplasm, in pear-shaped interneurons and in neuritic processes. In the neostriatum (caudate nucleus), decreased GAD67 immunostaining in the HIV infected subject was not as clear-cut but still was present (**e** vs **f**). The neurons in the globus pallidus also were stained less intensely in the HIV infected subject. Note that the number of stained neurons in the HIV infected subject is similar to the seronegative control (**g** vs **h**). Scale bars are 200 um in **a** and **b**, and 100 um in **c** – **f**

**Fig. 6:**
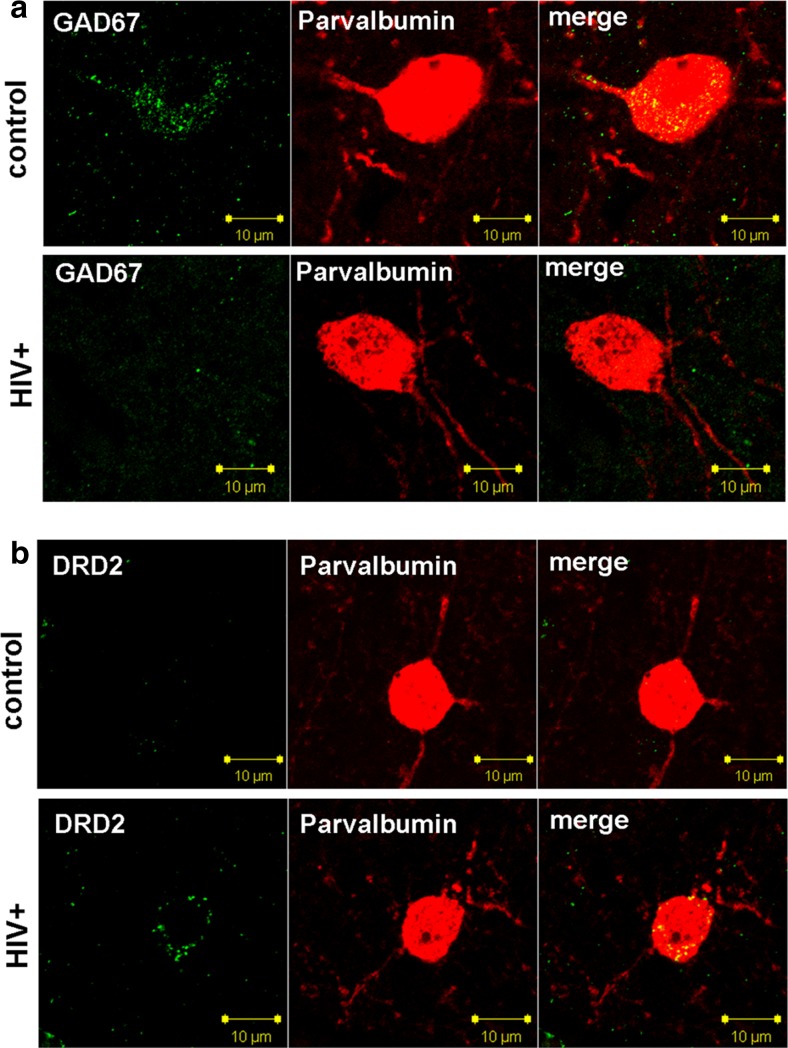
The six panels in **a** illustrate that loss of GAD67 staining in HIV infected patients does not correspond to the dropping out of interneurons. Immunofluorescence for interneuronal markers GAD67 and parvalbumin (PV) show that the cortical interneuron from the HIV-infected subject contains less intense GAD67 staining compared to the seronegative control. PV immunoreactivity of interneurons and neuritic processes is of equivalent intensity in these two specimens. The six panels in **b** illustrate that HIV-infected patients with HAND had high expression of dopamine receptor 2 (DRD2) in cortical interneurons, when interneuron from a seronegative patient lacks DRD2 staining. PV immunoreactivity of interneurons and neuritic processes is of equivalent intensity in these two specimens. Scale bars are 10 um

Western blotting for GAD67 (the protein product of *GAD1* transcript) showed that GAD67 expression was lower in 36 representative HIV infected subjects as compared to 12 uninfected subjects (Fig. 4). GAD67-immunostainining in the DLPFC further illustrated that protein staining intensity was lower in the neocortex of infected subjects (Fig. 5). Stain intensity in DLPFC was lowered diffusely in cell bodies and neural processes of the cortical laminae. Dual staining was performed to determine whether GAD67 and higher DRD2 protein expression were co-localized in the same neurons. Weak GAD67 staining hindered the effort to localize the two antigens in the infected subjects (not illustrated). When we stained for GABAergic cell markers that were more abundantly expressed, inhibitory interneurons in DLPFC often had strong expression of DRD2 in the HIV infected, but not in the seronegative control specimens (Fig. 6b). Dual staining for combinations of two different markers of inhibitory neuronal subpopulations confirmed that the loss of neocortical GAD67 staining occurred in viable interneurons. Examples are illustrated in Fig. 6a, which shows the lack of staining for GAD67 in numerous PV-stained inhibitory neurons of the HIV infected patients. In contrast, the seronegative controls had primarily PV-stained neurons that were positively stained for GAD67. The lack of GAD67 staining in the HIV infected patients was observed almost exclusively in interneurons with morphologically typical-appearing nuclear and perikaryal features.

## Discussion

This study elucidates the neurochemical, neuropathological, neurocognitive and neurovirological aspects of abnormally low expression of GABAergic markers in HIV-infected patients. Approaching these questions using tissues from a large number of patients imparted strong statistical power. Results with three separate GABAergic mRNAs were consistent, which provided added rigor to the experimental design. Our emphasis on the DLPFC was appropriate given that HAND and other diseases with abnormal GABAergic transmission have neuropsychological deficits that can be traced to abnormal output of the DLPFC (Iudicello et al. 2007; Woods et al. 2009). GABAergic neurotransmission is abnormal in many HIV-infected patients, as previously suggested (Masliah et al. 1995; Fox et al. 1997; Gelman et al. 2012a). Our data on brain virus replication show that the GABAergic anomaly is not correlated with the amount of replicating HIV-1 in the CNS. Further, the data show no significant association with HIVE, which is a neuropathology driven by replicating HIV-1 in the CNS. Also, the anomaly was not normalized in patients taking cART, which is a treatment that lowers viral replication. All of these observations indicate that reduced expression of GABAergic markers is not driven directly by the amount of replicating HIV-1 in the CNS or systemically. The lack of linkage with replicating HIV-1 as demonstrated herein is striking because it runs contrary to many conclusions suggested by prior work done using far fewer subjects (Masliah et al. 1995). Our conclusion regarding the lack of a direct role of HIV-1, while potentially counterintuitive and at-odds with prior suggestions draws support from the large patient sample. Our conclusion also is consistent with recent data showing that the neurovirological correlation between brain viral replication and HAND is not significant in most patients taking cART (Gelman et al. 2013).

### Neuroimmune Responses were Related to Abnormal Expression of GABAergic Markers

This and other studies show that several aspects pertaining to the neurobiology of HIV infection in humans do not appear to be controlled directly by HIV replication in the CNS (Gelman et al. 2012a; Gelman et al. 2013). Indeed, there is abundant evidence that host immune responses in the CNS may play a critical role in driving HAND in a manner that is partly independent of HIV replication (Glass et al. 1995). Consistent with that, we found that low GABAergic transcripts were significantly correlated with higher neuroimmune type markers in the brain including interferon response genes. These findings agree with reports which showed that the host immune response in the CNS (e.g., activation of brain macrophages and microglia), but not the concentration of HIV RNA *per se*, drove the development and progression of neurocognitive impairment (Glass et al. 1995; McArthur et al. 2010; Gelman et al. 2013). The fact that patients without encephalitis often had abnormal GABAergic transmission remains unexplained, yet is consistent with the fact that HAND also is highly prevalent in patients without encephalitis.

### Substance use History was not Related to Abnormal GABAergic Markers Expression

Drug addiction was common in this HIV-infected patient cohort and could have influenced synaptic transmission; 89 out of 204 HIV-infected patients with self-reports had current or past history of substance abuse. That rate is comparable generally to other HIV-infected populations in which lifetime prevalence of any substance use ranges from 23 to 56 % (Klinkenberg et al. 2004). In our cohort GABAergic expression was low in subjects with and without a history of drug dependence, and the two groups of subjects were not statistically different from each other. This result was somewhat unexpected because substance abuse often does result in the altered GABAergic neurotransmission. Altered expression of GABAergic mRNAs and proteins were reported for cocaine (Enoch et al. 2012), nicotine (Satta et al. 2008), methamphetamine (Zhang et al. 2006; Anneken et al. 2013), and morphine use (Sultana et al. 2010). These discrepancies are likely to be related to differences in experimental designs, as studies of substance abuse predominantly utilized animal models and acute dosing paradigms. The combined influence of substance abuse and HIV-1 on inhibitory system was studied in very few prior studies (Langford et al. 2003; Chana et al. 2006). It has been hypothesized that calbindin- and parvalbumin-expressing interneurons are selectively vulnerable to neurodegeneration in methamphetamine-using patients with HIVE. We suggest that the reason for our sharply contrasting conclusion (that these neurons do not undergo degeneration) is that we used multiple markers of inhibitory neurons to illustrate the lack of cell dropout. Other differences between this and previous studies include their use of smaller cohorts with highly restrictive inclusion and exclusion criteria, and the inherent differences between using postmortem human brain tissue versus animal models.

### The Neurovascular Unit is Linked to Abnormal Expression of GABAergic Markers

Brain gene array data have suggested that patients with HAND who are taking cART, but do not have high viral replication rates in the CNS or encephalitis, might harbor a disturbance in the neurovascular unit (NVU) (Gelman et al. 2012a). The components of the NVU include endothelial cells, astrocytic end feet, nerve endings and accessory cells that include pericytes, perivascular microglial cells, and macrophages (Ballabh et al. 2004). It is notable that the most significant association of reduced expression of GABAergic markers that we observed was with higher expression of the brain endothelial cell markers. Since the endothelial cells are in direct contact with blood plasma, our results suggest that changes in GABAergic transmission may be linked via the NVU to systemic changes in virally suppressed patients, such as persistent inflammation (Gelman 2015). One possible scenario is that increased expression of cell adhesion molecules and endothelial markers (e.g., VCAM-1, ICAM-1, PECAM1, and von Willebrand factor) facilitates the transmigration of infected immune cells across the BBB (Eugenin et al. 2006). Increased concentrations of inflammatory molecules and viral proteins in the brain tissue often result in activated astroglia, disrupted glutamate / glutamine cycle and lower rate of GADs expression (Janda et al. 2011).

### Loss of GABAergic Immunostaining Does not Reflect Neurodegeneration

Studies conducted before the era of cART suggested that HAND is a classical neurodegenerative disease that produces the loss of nonviable inhibitory interneurons. The validity of this suggestion as it pertains to cART-era brain specimens with HAND remains doubtful (Gelman and Moore 2011; Gelman 2015). This concept also has been challenged in patients with schizophrenia who have GABAergic disturbances (Benes et al. 1991). Our observations indicate that loss of GABAergic mRNAs and protein occurs in viable inhibitory interneurons. DLPFC specimens from HIV-infected patients generally had decreased GAD67 staining intensity across the cortical lamina, and the affected interneurons still expressed calretinin, parvalbumin or somatostatin, which are alternative markers expressed by GABAergic neurons. The affected neuronal profiles had the morphological characteristics of fully viable interneurons with normally proportioned cell bodies and nuclear profiles that were not suggestive of necrosis or apoptosis. Our results offer little or no support for the hypothesis that the abnormal GABAergic neural transmission in HIV infected brain specimens is caused by classical neurodegeneration. The data suggest instead that transcription of genes is down-regulated, or that the stability of mRNAs of rate limiting enzymes is lost. Such changes lie within the range of changes that can drive synaptic plasticity and accommodation to stress (Gelman et al. 2012b). Our suggested interpretation is compatible with results from patients with schizophrenia, mood disorders, and Parkinson’s disease, in whom neocortical *GAD1* mRNA is abnormally expressed and loss of inhibitory neurons is not observed (Akbarian et al. 1995; Volk et al. 2000; Lanoue et al. 2010). Our results also agree with a report showing that parvalbumin-expressing interneurons did not drop out in the neocortex of patients with HIVE (Masliah et al. 1992). Taken altogether, the GABAergic disturbance in HIV infected patients probably reflects synaptic plasticity and accommodation instead of classical neurodegeneration of a subpopulation of cells.

### Reduced Expression of GABAergic Markers is Linked to Worse Cognitive Performance in HIV-1

Down-regulated levels of *GAD1* mRNA and GAD67 protein are characteristic features of various neurocognitive and psychiatric disorders, including autism, bipolar disorder, epilepsy, and schizophrenia (Akbarian et al. 1995; Volk et al. 2000; Thompson et al. 2009). This study found that HIV-infected patients with and without neurocognitive impairment both had lower GABAergic mRNAs, but were not significantly different when compared to each other. Moreover, we did not find significant difference between GABAergic mRNAs concentrations in groups with various degrees of cognitive impairment. Thus, abnormal GABAergic transmission *per se* is not a promising candidate biomarker for the nosological diagnosis of HAND. We did find, however, that all three GABAergic mRNAs were correlated specifically with worse performance on verbal fluency tasking, which also is abnormal in patients with frontal lobe trauma, Parkinson’s disease, and Huntington’s disease (Millikin et al. 2004). Verbal fluency tasking has two distinct functional components – phonemic (letter) and semantic (category) fluency, each consisting of two subtasks – clustering and switching. More than 40 % of cases of impaired verbal fluency in HIV-infected patients have been linked to switching impairment (Millikin et al. 2004). Voxel-based lesion symptom mapping shows that phonemic switching is driven by the frontal lobes, whereas semantic switching relies on medial temporal lobes (Baldo et al. 2006). In HAND both semantic and phonemic fluency deficits are of similar magnitude, which suggests that frontal and temporal lobe functions both are involved. A key niche for future study is whether abnormal GABAergic transmission in the temporal lobes disproportionately impacts worse performance on tests of semantic switching.

### Abnormal Expression of GABAergic Markers is Interrelated with Other Neurotransmitter Transmission Systems

Using the same cohort of HIV infected patients, it was shown previously that failure to suppress the expression of dopamine receptor type 2 long isoform mRNA (*DRD2L*) in the DLPFC was associated with worse neurocognitive performance (Gelman et al. 2012b). The implied interpretation is that lower expression of *DRD2L* is a beneficial accommodation driven by higher presynaptic tone. The same report showed that enkephalinergic neural transmission also undergoes an accommodative type of decrease in many HIV infected brains. Both of those changes illustrate that synaptic plasticity could be central to the general mechanism of HAND. Abnormal expression of GABAergic genes was linked to changes associated with higher dopaminergic tone and lower enkephalinergic synaptic tone. These neurotransmitter systems interactions often could be observed histologically in tissue sections, as in our finding of higher expression of DRD2 in the interneurons lacking GAD67 immunoreactivity. These observations in HIV infected patients are consistent with reports from uninfected people that DRD2 is mostly expressed by large parvalbumin-expressing interneurons and small pyramidal neurons (Khan et al. 1998). Mechanistic studies indicate that dopamine stimulation of DRD2 and overexpression of DRD2 decreases GABA synthesis in inhibitory interneurons (Seamans et al. 2001). In turn, it is suggested that the lower GABA inhibitory currents lead to a shift toward excitation in local inhibitory microcircuits, which results in uncontrolled spread of activation and a decreased signal-to-noise ratio. At the behavioral level, the clinical manifestations of inhibitory circuit dysfunction include impaired working memory, planning and executive functions, slowed thinking, and word finding difficulties. All these features are characteristic neuropsychological findings in patients with schizophrenia and HAND (Seamans and Yang 2004; Iudicello et al. 2007; Woods et al. 2009). Thus, higher dopaminergic tone is one potential mechanism that could diminish GABAergic transmission in HIV-infected patients. While the differing types of neurotransmitter systems exhibit substantial interrelationships to each other, they do not necessarily drive the same functional deficit. For example, the GABAergic anomaly in DLPFC relates primarily to worse performance on tasks of verbal fluency (Table 1, discussed above), whereas defective dopaminergic tone in the DLPFC (Gelman et al. 2012b) was related to a broader spectrum of neurocognitive dysfunction (Seamans and Yang 2004). In contrast to those systems, abnormal enkephalinergic transmission (low *PENK* mRNA) showed little relationship to any of the neurocognitive functions that were examined. A highly complex picture emerges that involves shifting of multiple interconnected neurotransmitter systems, each having implications regarding particular aspects of abnormal neurocognitive function in HAND.

In sum, this neurochemical survey shows that GABAergic markers are abnormally low in the frontal neocortex of a substantial proportion of HIV infected patients. The differences were likely to have been regulated transcriptionally versus being the results of pathological neurodegeneration. Worse performance on tasks of verbal fluency was related significantly to lower frontocortical GABAergic marker expression, but other types of tasking were not related to it. Neither brain HIV replication nor encephalitis were significantly associated with GABAergic abnormalities. Brain markers associated with activated neuroimmunity and heightened endothelial cell activity both were linked significantly to low GABAergic marker expression, which suggests involvement of systemic immunity and the NVU.

## Electronic Supplementary Material

Below is the link to the electronic supplementary material.

Fig. S1 (GIF 99 kb)

(TIFF 2499 kb)

Fig. S2 (GIF 16 kb)

(TIF 276 kb)

Fig. S3 (GIF 114 kb)

(TIFF 3046 kb)

Table S1 (DOCX 16 kb)

Table S2 (DOCX 16 kb)
