# Supplementary figures and images for: Neuropsychological, Neurovirological and Neuroimmune Aspects of Abnormal GABAergic Transmission in HIV Infection
Source: J Neuroimmune Pharmacol. 2016 Jan 30;11:279–93. doi: 10.1007/s11481-016-9652-2 (PMC4848342; doi:10.1007/s11481-016-9652-2)

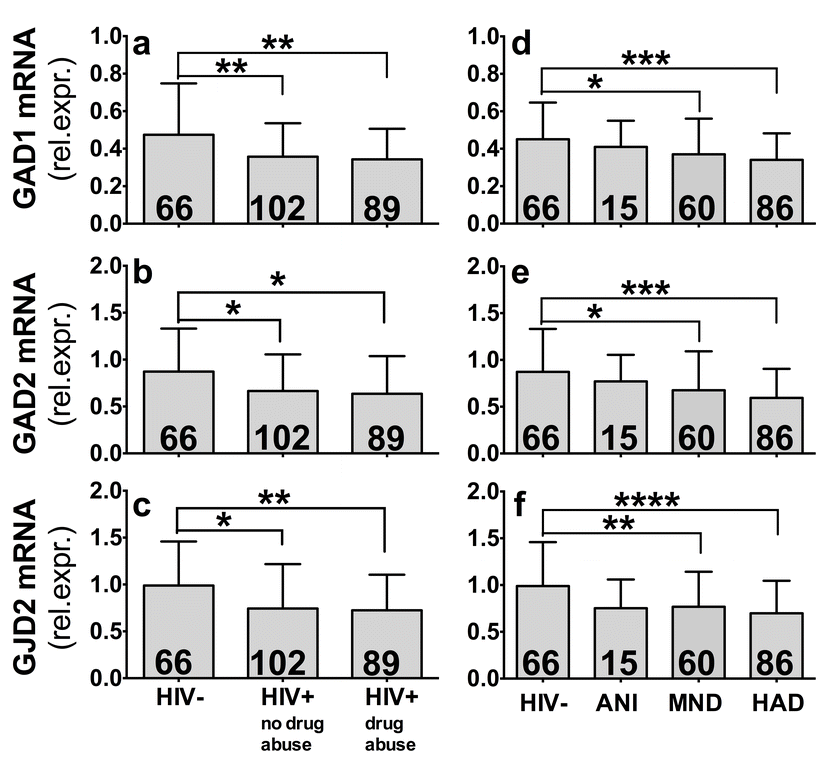

Supplement: Supplementary file 1 — (GIF 99 kb) [file 11481_2016_9652_Fig7_ESM.gif]

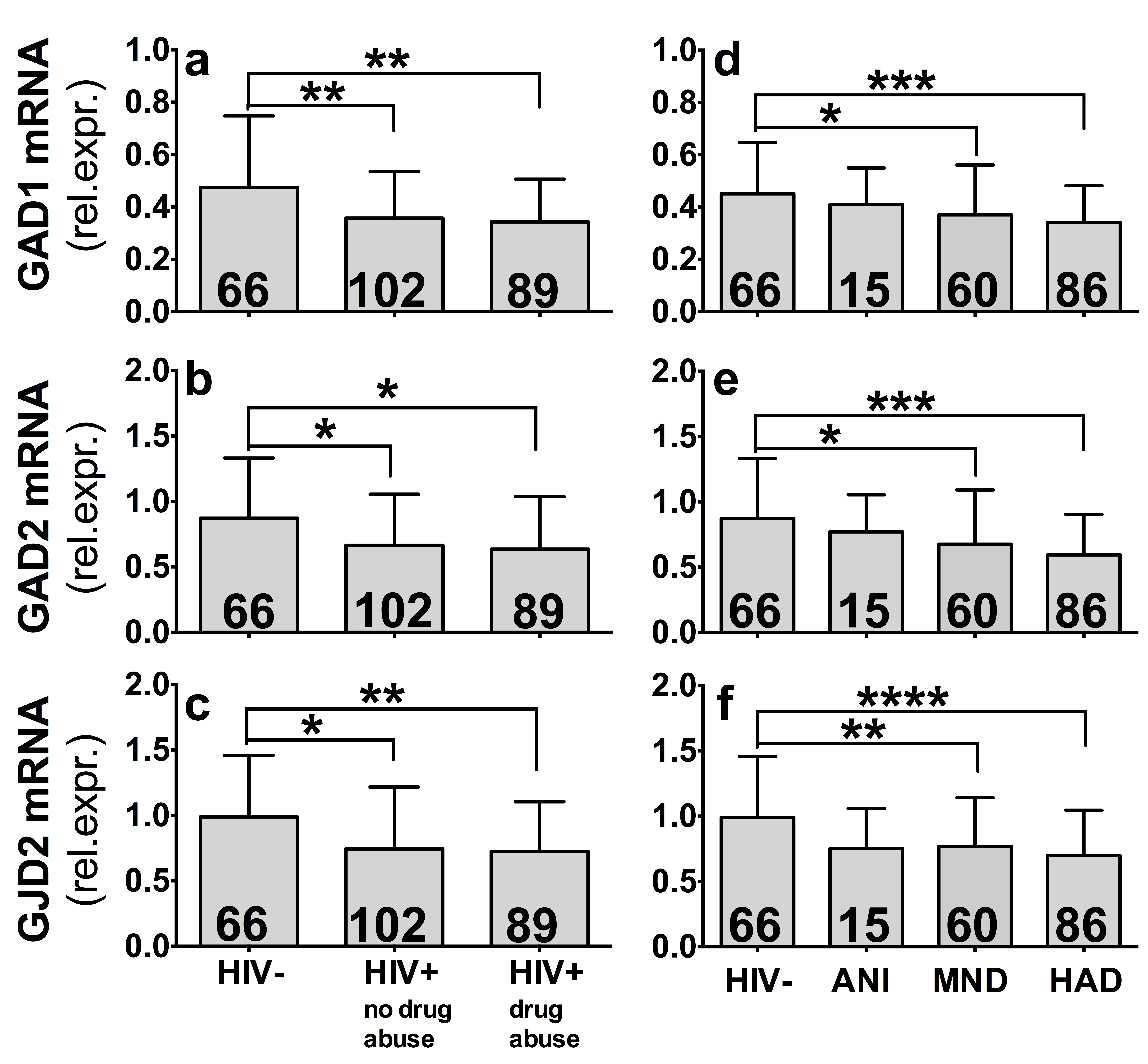

Supplement: Supplementary file 2 — (TIFF 2499 kb) [file 11481_2016_9652_MOESM1_ESM.tiff]

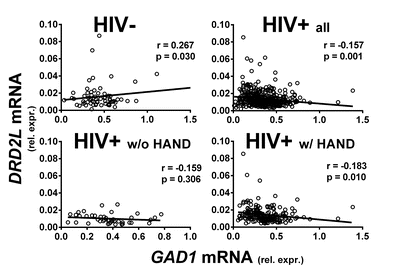

Supplement: Supplementary file 3 — (GIF 16 kb) [file 11481_2016_9652_Fig8_ESM.gif]

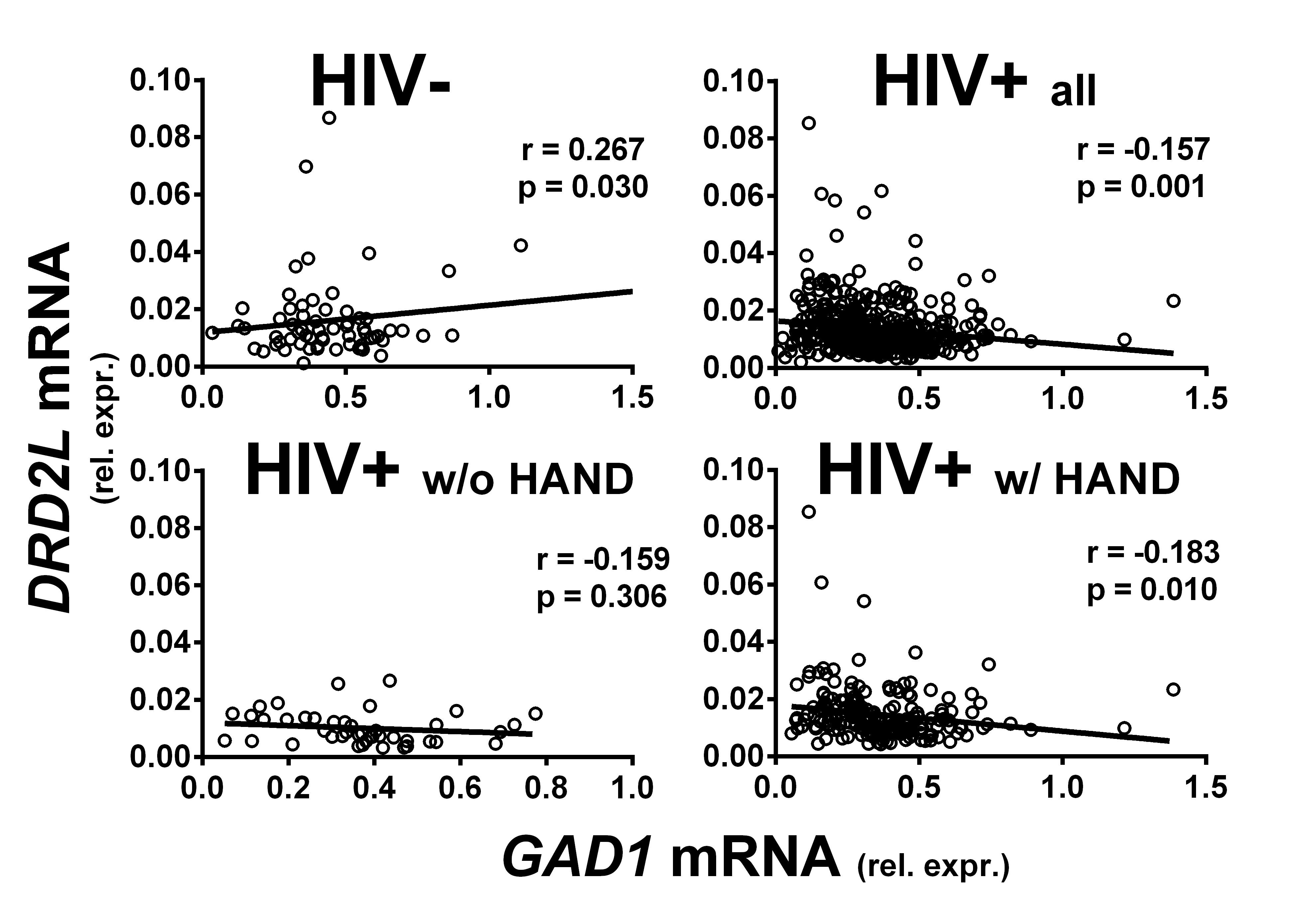

Supplement: Supplementary file 4 — (TIF 276 kb) [file 11481_2016_9652_MOESM2_ESM.tif]

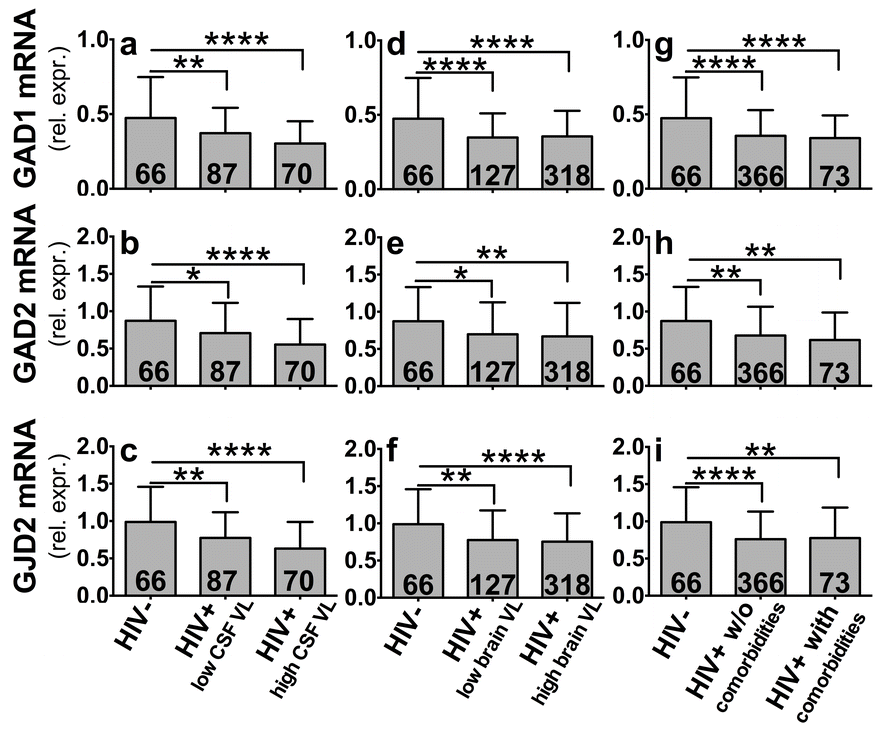

Supplement: Supplementary file 5 — (GIF 114 kb) [file 11481_2016_9652_Fig9_ESM.gif]
