# Supplementary material for: Neuropsychological, Neurovirological and Neuroimmune Aspects of Abnormal GABAergic Transmission in HIV Infection
Source: J Neuroimmune Pharmacol. 2016 Jan 30;11:279–93. doi: 10.1007/s11481-016-9652-2 (PMC4848342; doi:10.1007/s11481-016-9652-2)
Supplement: Supplementary file 7 — (DOCX 16 kb) [file 11481_2016_9652_MOESM4_ESM.docx]

| **Table S1** HIV positive subjects with and without HIV encephalitis or HIV associated neurocognitive disorders and seronegative controls | | | | | | | | | |
| --- | --- | --- | --- | --- | --- | --- | --- | --- | --- |
| Characteristic | HIV infection | | *p-value* | HIV encephalitis |  | *p-value* | HIV associated dementia | | *p-value* |
|  | HIV- | HIV+ | HIV- vs HIV+ | HIVE- | HIVE+ | HIVE- vs HIVE+ | no HAND | HAND | no HAND vs HAND |
| Number of subjects | 63 | 449 | N/A | 357 | 92 | N/A | 43 | 161 | N/A |
| Age (years, mean ± stdev) | 44.9 ± 11.4 | 42.6 ± 9.6 | 0.0782 | 42.9 ± 10 | 41.3 ± 7.7 | 0.139 | 48 ± 8.1 | 43.4 ± 12.2 | 0.003 |
| Gender (male/female) | 49/ 14 | 387 / 62 | 0.113 | 302/55 | 85/7 | 0.053 | 39/4 | 129/32 | 0.106 |
| Race (W/B/A/O) | 39/18/0/6 | 271/149/6/23 | 0.431 | 221/114/6/16 | 53/32/0/7 | 0.341 | 35/7/0/1 | 105/40/3/13 | 0.186 |
| Hispanic or Latino (yes/no) | 16/47 | 87/362 | 0.356 | 64/293 | 23/69 | 0.126 | 37/ 6 | 35/126 | <0.001 |
| Postmortem interval (hours, mean ± stdev) | 15.5 ± 11.7 | 14.5 ± 14.7 | 0.6683 | 14.2 ± 13.5 | 15.6 ± 18.8 | 0.426 | 21 ± 27.6 | 13 ± 12.2 | 0.006 |
| Log10 Plasma HIV RNA (copies/ml, mean ± stdev) | N/A | (n=258) 4.3 ± 1.4 | N/A | (n=217) 4 ± 1.6 | (n=47) 5.1 ± 0.9 | p < 0.001 | (n=39) 3.6 ± 1.8 | (n=136) 4.3 ± 1.5 | 0.023 |
| Log10 CSF HIV RNA (copies/ml, mean ± stdev) | N/A | (n=181) 2.9 ± 1.5 | N/A | (n=163) 2.6 ± 1.3 | (n=28) 4.2 ± 1.7 | p < 0.001 | (n=32) 2.5 ± 1.4 | (n=90) 2.8 ± 1.3 | 0.391 |
| Blood CD4+ lymphocyte count (cells/mm^3^, mean ± stdev) | N/A | (n=274) 106.5 ± 162 | N/A | (n=222) 119.4 ± 174.9 | (n=52) 51.5 ± 64.1 | 0.006 | (n=41) 158.7 ± 177.7 | (n=139) 103.1 ± 176. | 0.078 |
| HAART treatment (yes/no) | N/A | 131/318 | N/A | 249/108 | 69/23 | 0.323 | 43/0 | 147/14 | 0.045 |
| ^a^ HIV-, Human Immunodeficiency Virus seronegative; HIV+, seropositive | | | | | | | | | |
| ^b^ HIVE, HIV encephalitis | | | | | | | | | |
| ^c^ HAND, HIV associated neurocognitive disorders | | | | | | | | | |
| ^d^ mean±standard deviation, Student’s t-test | | | | | | | | | |
| ^e^ M/F, Male/female | | | | | | | | | |
| ^f^ Chi-square | | | | | | | | | |
| ^g^ B/W/A/O, Black/White/Asian/Other | | | | | | | | | |
| ^h^ N/A, not applicable | | | | | | | | | |
