# Supplementary material for: Neuropsychological, Neurovirological and Neuroimmune Aspects of Abnormal GABAergic Transmission in HIV Infection
Source: J Neuroimmune Pharmacol. 2016 Jan 30;11:279–93. doi: 10.1007/s11481-016-9652-2 (PMC4848342; doi:10.1007/s11481-016-9652-2)
Supplement: Supplementary file 8 — (DOCX 16 kb) [file 11481_2016_9652_MOESM5_ESM.docx]

| **Table S2** GABAergic transcripts correlated with HIV-1 replication and CD4+ lymphocyte count | | | | | | | |
| --- | --- | --- | --- | --- | --- | --- | --- |
|  | | ***GAD1* mRNA** | | ***GAD2* mRNA** | | ***GJD2* mRNA** | |
| ***HIV+, all*** | n | *r* | *p* | *r* | *p* | *r* | *p* |
| log brain VL | 447 | -0.0021 | 0.9647 | -0.0661 | 0.1630 | -0.0017 | 0.9714 |
| log blood VL | 264 | -0.1067 | 0.0836 | -0.1372 | 0.0258* | -0.0949 | 0.1240 |
| log CSF VL | 191 | -0.1250 | 0.0849 | -0.1584 | 0.0286* | -0.1801 | 0.0127* |
| Blood CD4 cell count | 274 | 0.0414 | 0.4950 | 0.0661 | 0.2756 | 0.1163 | 0.0545 |
| ***HIV+, no HIVE*** | | | | | | | |
| log brain VL | 355 | -0.1045 | 0.0491* | -0.0926 | 0.0815 | -0.0311 | 0.5592 |
| log blood VL | 217 | -0.1340 | 0.0487* | -0.1502 | 0.0269* | -0.1123 | 0.0990 |
| log CSF VL | 163 | -0.1426 | 0.0694 | -0.1642 | 0.0362* | -0.2077 | 0.0078* |
| Blood CD4 cell count | 222 | 0.0468 | 0.4878 | 0.0701 | 0.2984 | 0.1368 | 0.0417* |
| ***HIV+, HIVE*** | | | | | | | |
| log brain VL | 92 | 0.1544 | 0.1417 | 0.0864 | 0.4128 | 0.2188 | 0.0361* |
| log blood VL | 47 | -0.0053 | 0.9718 | 0.0124 | 0.9341 | 0.1244 | 0.4048 |
| log CSF VL | 28 | -0.1284 | 0.5149 | -0.0918 | 0.6422 | -0.0050 | 0.9799 |
| Blood CD4 cell count | 52 | 0.1289 | 0.3624 | 0.0767 | 0.5889 | -0.0580 | 0.6830 |
| *Asterisk denotes statistically significant *p* value. Brain HIV-1 RNA and GABAergic transcripts were measured in the frontal neocortex | | | | | | | |
| HIV human immunodeficiency virus type 1, HIVE HIV encephalitis, *r* correlation coefficient, CSF cerebrospinal fluid | | | | | | | |
